# The Challenge–Hindrance–Threat Appraisal Framework and the Differential Effects on Employees’ Work Well-Being and Behaviors

**DOI:** 10.3390/bs14090734

**Published:** 2024-08-23

**Authors:** Qin Chen, Mengfan Chen, Lin Lin, Xinwen Bai

**Affiliations:** 1 CAS Key Laboratory of Behavioral Science, Institute of Psychology, Chinese Academy of Sciences, Beijing 100101, China; bettycccq@sohu.com; 2 Department of Psychology, University of Chinese Academy of Sciences, Beijing 100049, China; 3 School of Business, Central University of Finance and Economics, Beijing 100081, China; 2023110147@email.cufe.edu.cn (M.C.); linlin@cufe.edu.cn (L.L.)

**Keywords:** work stress, challenge appraisal, hindrance appraisal, threat appraisal, work well-being, work behavior

## Abstract

Considering the current economic and employment landscape, marked by high levels of uncertainty and challenges, this study introduces the challenge–hindrance–threat appraisal (CHTA) framework to examine workplace stress, with a particular focus on the often-overlooked role of threat appraisal. Across three studies using independent samples and multi-wave survey data, our research provides evidence supporting the CHTA framework and reveals the differential effects of three types of stress appraisals on the work-related well-being and behaviors of individuals. Specifically, Study 1 establishes the three-factor structure of CHTA, confirming its robust psychometric properties in each appraisal dimension. Study 2 demonstrates that threat appraisals offer unique insights into stress-related outcomes, beyond challenge and hindrance appraisals. Study 3 reveals that challenge appraisals foster learning behavior through enhancing work engagement and reduce turnover intentions through alleviating emotional exhaustion. Conversely, threat appraisals escalate turnover intentions through intensifying emotional exhaustion. Interestingly, hindrance appraisals exhibit no significant effect on either work engagement or emotional exhaustion, precluding any indirect influence on learning behavior or turnover intentions. In conclusion, this research underscores the importance of differentiating between the three types of cognitive appraisals of stress—namely, challenge, hindrance, and threat appraisals—in stress management in order to enhance employee well-being and organizational effectiveness.

## 1. Introduction

Stress arises when individuals perceive the demands of events or environmental stimuli to exceed their capacity to manage, alleviate, or modify those demands [1], and stressors refer to the events and stimuli that elicit subsequent reactions [2]. Work stress has long been and continues to be a significant challenge for organizations and employees worldwide [3]. For example, the American Psychological Association’s 2023 Work in America Survey warned that workplace stress has reached a concerning level, with approximately two-thirds of employees indicating that they had experienced work-related stress in the month preceding the survey. Furthermore, more than half of the respondents reported substantial negative consequences as a result of work-related stress [4]. In the United Kingdom, according to the UK Health and Safety Executive’s summary statistics for 2023, work-related stress, depression, or anxiety accounted for nearly 50% of the 1.8 million reported cases of work-related ill health in the past 12 months [5]. In China, Gallup’s State of the Global Workplace 2024 report found that 53% of respondents reported experiencing stress for a significant portion of the preceding day, a percentage well above the global average of 41% and ranking China in the top 17 among the more than 160 countries covered by the survey [3]. Given its ubiquity and costs, it is not surprising that existing research on work stress has focused primarily on its adverse effects [6,7] and aimed to develop interventions to reduce stress and/or mitigate its negative consequences [8].

However, scholars who adopt a psychological perspective have argued that stress does not always result in negative effects; instead, the effects of stress largely depend on how individuals perceive it and the resources available for coping [7,9]. Researchers in the field of workplace stress have developed various theoretical models. One influential framework is Lazarus and Folkman’s transactional theory of stress, also known as the cognitive appraisal theory of stress [1]. According to this model, stress is not solely caused by external stressors but rather arises from how individuals appraise them. When faced with a stressor, individuals engage in primary appraisal to determine whether it is irrelevant, beneficial, or stressful. If deemed stressful, the stressor is further appraised as a challenge or a threat. Challenge appraisals occur when individuals perceive the stressor as an opportunity for growth or mastery, leading to optimism and the expectation of positive outcomes. Conversely, threat appraisals occur when individuals perceive potential harm or loss, resulting in anxiety and fear. Secondary appraisal involves evaluating available coping resources and options. In summary, this model highlights the subjective nature of stress and emphasizes that the impact of stressors is primarily determined by how individuals cognitively appraise them.

Another significant framework in the study of work stress is the challenge–hindrance approach [10,11]. This framework categorizes workplace stressors as either challenge stressors or hindrance stressors. Challenge stressors, such as high workload and increased responsibility, are demanding but offer potential for personal growth, learning, and goal achievement. In contrast, hindrance stressors, such as red tape and role conflict, impede the attainment of goals and are perceived as constraints or threats without potential gains. These stressors have different effects on outcomes, with challenge stressors being associated with positive outcomes such as higher job satisfaction and performance, while hindrance stressors are correlated with negative outcomes such as burnout and strain. Recent research has suggested that the process of appraising stressors is complex [12,13]. Stressors can be simultaneously perceived as both challenges and hindrances, varying over time and across individuals. This indicates that strictly classifying stressors as either challenges or hindrances oversimplifies the cognitive appraisal process.

Surprisingly, although both models emphasize the necessity of a nuanced understanding of stressor appraisal and have been extensively utilized in stress research, an overarching framework to comprehensively integrate the empirical findings is lacking. While each model emphasizes the significance of challenge appraisal and posits that it is different from hindrance or threat appraisal, it remains unclear whether the latter two can be differentiated from each other. Although preliminary research has indicated that threat, hindrance, and challenge appraisals can exist independently [14,15], more studies are needed to conceptually and empirically clarify the nature of different cognitive appraisals. Another notable limitation in the extant stress appraisal literature lies in the assumption that cognitive appraisal is inherently associated with a certain type of stressor. According to this view, a stressor should be exclusively classified as a challenge, hindrance, or threat stressor [2,15]. Furthermore, individuals tend to appraise challenge stressors as challenges, hindrance stressors as hindrances, and threat stressors as threats [15,16]. This view is problematic, as increasing research has demonstrated that this is not always the case [17,18]. Instead, individuals may simultaneously appraise the same stressor from multiple perspectives, rather than adhering to a specific appraisal [19,20,21,22].

To address these limitations, we integrate these two frameworks and propose the challenge–hindrance–threat appraisal (CHTA) model in order to systematically examine how workplace stress is appraised and how stress appraisals shape an individual’s work well-being and behaviors. In this newly proposed model, we posit that the same stressor can be simultaneously perceived as a challenge, a hindrance, and/or a threat; consequently, different cognitive appraisals of the same stressor yield different effects on the affective states and behaviors of employees. We plan to conduct a series of independent studies to establish and cross-validate our CHTA model. Specifically, Study 1 involves construction of the three-factor model of cognitive appraisals and preliminarily tests its factor structure. Study 2 proceeds to cross-validate the three-factor structure and test its psychometric properties in terms of convergent, discriminant, and predictive validities. Finally, Study 3 aims to investigate the differential effects of three appraisals on both proximal and distal outcomes. In this way, our study strives to develop a comprehensive theoretical framework of stress appraisals, deepening our knowledge on how stress appraisals can shape employee experiences and behaviors.

## 2. Theories and Literature Review

### 2.1. The Challenge–Threat Appraisal Framework

Individuals may exhibit different cognitive and appraisal responses to identical stressors. To explore how individual characteristics influence the variability in the stressor–outcome relationship, Lazarus and Folkman proposed the cognitive appraisal theory of stress, also known as the transactional model of stress [1]. This model highlights the process of appraising stimuli and stress, which refers to an individual’s perception of stress. The model assumes that individuals consistently evaluate their environment based on its impact on their well-being (primary appraisal) as well as potential coping resources and options (secondary appraisal) [1]. Primary stress appraisals can take three different forms: harm or loss already experienced, the threat of future harm or loss, or the possibility for mastery and gain (known as challenges). Challenge appraisals prioritize the mastery of needs, overcoming obstacles, and personal growth; conversely, threat appraisals involve assessing expected harm or loss to oneself [23]. It is important to note that threat and challenge appraisals are not opposite ends of a spectrum and, thus, are not necessarily mutually exclusive. In fact, they can occur simultaneously or interchangeably [1]. Consistent with the core premise of this model, cumulative evidence indicates that it is the individual’s cognitive appraisal of the stressor, rather than the stressor itself, that determines their emotional, attitudinal, and behavioral responses and consequences [24]. If individuals perceive a stressor to be threatening and lack the necessary resources to mitigate its detrimental effects, it can lead to negative outcomes, including diminished well-being and a tendency towards withdrawal behaviors. On the other hand, when individuals view the stressor as challenging and possess ample resources for coping, they tend to exhibit positive reactions, thereby sustaining a higher level of well-being and demonstrating proactive work behaviors.

### 2.2. The Challenge–Hindrance Appraisal Framework

The challenge–hindrance stressor framework has been widely utilized in empirical research for more than two decades, since its initial introduction by Cavanaugh et al. [10]. Representing one of the key theoretical models of stress, this framework categorizes stressors into two dimensions: challenge stressors and hindrance stressors. The former refers to work-related demands or environments that offer potential gains for individuals, while the latter refers to work-related demands or environments that tend to constrain or interfere with an individual’s work achievement, often unrelated to their potential gains [10]. Job stressors, including workload, time pressure, job responsibility, and job complexity, are usually categorized as challenge stressors as they can potentially enhance employees’ personal growth and goal achievement [2,11,25]. Other stressors, such as role ambiguity and conflict, bureaucratic procedures, and job insecurity, are labeled as hindrance stressors as they usually constrain personal development and work-related accomplishment [2,11,25]. As extant empirical evidence has consistently indicated, challenge stressors mostly yield positive effects, while hindrance stressors mostly lead to negative effects in terms of various affective, attitudinal, and behavioral outcomes, including organizational loyalty and turnover intention [26], job satisfaction [2], life satisfaction [27], citizenship behavior [28], and job performance [29].

Recent research has suggested that the process of appraising stressors is more complex than what is assumed in this framework [12,13]; for example, stressors initially considered as hindrances, such as role ambiguity and role conflict, may actually be appraised as challenging [13,19]. Similarly, those initially considered as challenge stressors (e.g., workload) can also be appraised as both challenging and a hindrance [13]. More importantly, the effects of these stressors on the attitudinal and behavioral outcomes of employees are mediated through cognitive appraisals [13]. All of this evidence indicates that any stressor can be simultaneously appraised as both a challenge and hindrance, and strictly classifying stressors as either challenges or hindrances is an oversimplification of the cognitive appraisal process [30]. Therefore, the challenge–hindrance stressor framework should be refined and extended to explicitly incorporate the cognitive appraisals of individuals [30]. In this regard, scholars who have long adopted the challenge–hindrance dichotomy have begun to emphasize the important role of cognitive appraisals as mechanisms that transmit the effects of challenge and hindrance stressors to outcomes [16,25].

### 2.3. An Integrative Framework: The Challenge–Hindrance–Threat Appraisal Framework

While both of the above-mentioned models highlight the importance of the challenge appraisal of stress, they differ from each other in considering hindrance versus threat appraisals. Specifically, hindrance appraisal is defined in challenge–hindrance stressor research as “an individual’s subjective interpretation that demands have a potential to result in loss, constraints, or harm” [31] (p. 1039). Similarly, threat appraisal is defined as “harms or losses that have not yet taken place but are anticipated” in the transactional model of stress [1] (p. 32). Given that hindrance and threat appraisals are theoretically defined in similar ways, it remains unclear whether and to what extent they are distinguishable from each other.

Tuckey et al. are pioneers in this direction [14,15]. Drawing on Lazarus and Folkman’s (1984) [1] conceptualization of threat appraisal, Tuckey et al. (2015) [15] introduced the concept of threat work stressors, defining them as work-related demands or situations that are often directly related to physical harm or loss, as opposed to challenge and hindrance stressors. In the organizational context, threat stressors include workplace bullying and harassment [32], client-related social stressors [33], and abusive supervision [34], among others. These stressors reflect the process by which the stressor directly leads to physical harm or loss (threat) rather than an impediment to the achievement of goals (hindrance) [14].

Tuckey et al. provided some empirical evidence for distinction among the three types of stressors and appraisals. For instance, in their qualitative study involving interviews and focus groups with retail employees [14], they found that employees were aware of which stressor could pose a threat (threatening the self or causing physical harm and loss), a hindrance (preventing goal achievement), or a challenge (providing opportunities for personal growth). Taking role ambiguity—which is often viewed as a hindrance—as an example, participants did not just view it as a hindrance to work achievement; rather, they perceived it as a threat as it endangered their occupational self-efficacy, self-confidence, and self-esteem. These findings were evidenced in another quantitative study that consisted of a two-wave questionnaire and a diary survey over three consecutive working days [15]. On the one hand, the two-wave questionnaire survey of 609 retail workers lent support to the three-factor structure of challenge–hinder–threat for stressors. On the other hand, their diary study of 207 workers showed that challenge, hindrance, and threat appraisals were uniquely associated with affective states and well-being outcomes.

It is worth noting that, although Tuckey et al. deliberately make distinctions among the three appraisals, they anchor a specific type of appraisal to a single pre-categorized stressor (e.g., challenge appraisal is most likely triggered by a challenge stressor) [15]. As suggested in recent research, the view that a certain stressor will only trigger a particular appraisal might not be true. On the one hand, a large body of research has suggested that individuals may evaluate the same stressor in different ways [35,36]; for example, performance pressures may be perceived by individuals as a challenge, causing them to increase their work engagement and present higher task proficiency and citizenship behaviors, or as a threat, eliciting exhaustion and incivility [37]. On the other hand, the same stressor may elicit challenge, hindrance, and even threat appraisals from individuals [19,20,21,22]. Research suggests that pay for performance, workload, role conflict, role ambiguity, developmental work experiences, and empowered leadership may all simultaneously trigger challenging and hindrance appraisals in individuals [13,35,36].

In the current study, building on and extending the work of Tuckey et al. [14,15], we propose an integrative framework in which three types of cognitive appraisals—namely, challenge, hindrance, and threat appraisals—are distinct from each other. A key premise of our model is that individuals can simultaneously appraise the same stressor as a challenge, a hindrance, and/or a threat. Guided by this model, we explore the extent to which different cognitive appraisals of the same stressor will yield different effects on an employee’s affective states and behavior.

### 2.4. Preliminary Evidence for Differential Effects of Challenge, Hindrance, and Threat Appraisals

Since the genesis of the challenge–hindrance framework, subsequent meta-analyses have shown that challenge stressors mostly have a net positive impact on a variety of affective, attitudinal, and behavioral outcomes, whereas hindrance stressors mostly yield a negative impact [2,11,16]. Threat, on the other hand—although central to Lazarus and Folkman’s (1984) transactional model—has not received as much attention as challenge and hindrance stressors. Existing research has suggested that threat stressors cause higher psychological distress, emotional exhaustion, and burnout and lower job dedication and task performance in employees [38,39]. It is clear that, in the available literature, hindrances and threats are indistinguishable from each other in terms of outcomes.

Although cognitive appraisals are considered central to the challenge–hindrance framework, it was not until the study of Webster, Beehr, and Love (2011) that researchers began to measure both challenge appraisal and hindrance appraisal of work stressors [13]. At the same time, threat appraisals mentioned in the transactional model have received limited attention [14,15]. As mentioned earlier, it is essential to distinguish cognitive appraisals of stress from stressors and to emphasize the important role of cognitive appraisals as a mechanism through which the effects of the three stressors are transmitted to outcomes [16,25].

With this in mind, we focused on reviewing the differentiated outcomes of the three appraisals. Empirical evidence suggests that challenge, hindrance, and threat appraisals may have differential effects on both proximal and distal outcomes. Proximal outcomes refer to immediate emotional responses, work-related attitudes, and well-being. Research within the challenge–hindrance framework reveals that challenge appraisals have a positive relationship with activated positive affect, whereas hindrance appraisals correlate with the activation of negative emotions such as anger [12]. Furthermore, challenge appraisals are consistently found to be positively associated with work engagement [40], work-related flow [41], work dedication [15], vigor [14], motivation to work [42], organization-based self-esteem, and perceptions of meaningful work [43]. In contrast, hindrance and threat appraisals have a negative impact on attitudes and states in work, such as emotional exhaustion, job dissatisfaction [15], self-regulation depletion [37], burnout [40], venting [12], and job dissatisfaction [13]. Additionally, threat appraisals have been found to exert a unique impact on cognitive depletion and emotional exhaustion, suggesting their distinct role in shaping proximal outcomes [44]. Several studies adopting the challenge–hindrance framework also demonstrate differential effects on distal outcomes, including behaviors and job performance. For example, challenge appraisals show a positive relationship with increased task performance [12,31], task proficiency, organizational citizenship behavior [37], and creative performance [45]. Conversely, hindrance appraisals have a negative impact on job performance [12,13,46] and increase turnover intention [13].

As mentioned earlier, existing research conducted under the challenge–threat and challenge–hindrance frameworks has yet to provide direct evidence to distinguish between hindrance and threat appraisals. Indeed, there have only been a few studies using the challenge–hindrance–threat framework that provide evidence for the differential effects of the three appraisals (especially between hindrance and threat appraisals) [15,45]. Among them, Tuckey et al. [15] examined the differential impact of the three types of appraisals on affect and found that challenge appraisals of employees were associated with activated positive affect, hindrance appraisals with anxiety and anger, and threat appraisals with fatigue. Espedido and Searle [45] focused on creativity in the workplace and found that challenge appraisals were positively and hindrance appraisals negatively associated with both fluency and flexibility, but threat appraisals were positively related to fluency and negatively related to flexibility. Due to the lack of sufficient attention to the challenge–hindrance–threat appraisals of the same stressor, it remains unanswered whether the three appraisals will have differential effects on proximal and distal outcomes.

## 3. Current Research

To fill this important gap, our current study aims to firstly establish and cross-validate the challenge–hindrance–threat appraisal (CHTA) model and then to examine the differential effects of challenge, hindrance, and threat appraisals on both proximal and distal outcomes. We plan to conduct a series of independent studies to do so. Specifically, Study 1 aims to establish a three-factor structure for the integrative framework. Through obtaining another independent sample, Study 2 aims not only to cross-validate the challenge–hindrance–threat appraisal factor structure but also to test its psychometric properties in terms of convergent, discriminant, and predictive validity. In doing so, Study 2 helps to create an initial nomological network for the three types of appraisals. Finally, utilizing a three-wave survey design, Study 3 aims to investigate the extent to which the three appraisals are uniquely associated with the work states (i.e., work engagement and emotional exhaustion) and behavioral outcomes (i.e., learning behaviors and turnover intentions) of employees. Through employing a multi-sample strategy, the current study has the opportunity to establish and cross-validate the integrative framework and reveal the unique effects of each appraisal on workplace outcomes. Table 1 presents an overview of our three studies.

## 4. Study 1: Factor Structure

The objective of Study 1 was to generate measurement items for the CHTA framework as well as to initially examine the factor structure using confirmatory factor analysis (CFA).

### 4.1. Methods

#### 4.1.1. Participants and Procedures

This study recruited 224 working adults in China using Credamo (www.credamo.com), a reliable Chinese online data-collection platform similar to Qualtrics that is commonly used to recruit participants in China [47,48]. Anonymity in the survey was ensured by the platform through the automatic generation of unique user IDs for each participant, guaranteeing the confidentiality of their responses. Prior to the survey, participants provided informed consent through yes/no screening questions. They were informed that the survey would be anonymous and that they could withdraw at any time. The survey took an average of 10 min, and two instructed response items were included to discourage careless responses [49]. One such item was phrased as “This is an attention check question, please select the option ‘Strongly Disagree’”. At the conclusion of the survey, the participants were briefed, thanked, and compensated for their participation. Sixty-eight participants who either did not complete the survey or failed the attention checks were removed, resulting in a total of 156 participants in the valid sample. The participants had a mean age of 32.22 years (*SD* = 7.06), ranging from 21 to 56 years, and 64.7% were female.

#### 4.1.2. Measures

For the cognitive appraisals of stress, this study employed a strategy similar to that of Webster et al. (2011) [13]. First, this study presented text regarding the current economic and employment situation in China as a stressor. Second, in order to ensure that respondents interpreted the terms challenge, hindrance, and threat in a manner consistent with theory, we explicitly provided them with definitions that were consistently applied in earlier studies [10,12]. Specifically, challenge appraisal was defined as the perception of a challenging feeling where, although situations are deemed stressful, they also serve as motivation for personal growth and goal achievement. Hindrance appraisal was defined as the perception that progress at work was hindered in a stressful situation, making goal attainment difficult. Threat appraisal was defined as the experience of compromised interests and doubts about self-worth in stressful situations. Finally, participants were asked to rate their agreement with the challenge, hindrance, and threat appraisal items using a 5-point Likert-type scale ranging from 1 (strongly disagree) to 5 (strongly agree) for each appraisal. Scales for challenge, hindrance, and threat appraisals used in Studies 1, 2, and 3 can be found in Appendix A.

*Challenge Appraisal*. To assess challenge appraisal, we used the four-item measure developed by Searle and Auton (2015) [12]. A sample item was “The current economic and employment situation would help me to learn a lot”. The Cronbach’s alpha for this measure in our study was 0.85.

*Hindrance Appraisal*. Consistent with the challenge appraisal, the hindrance appraisal measure consisted of four items from Searle and Auton’s appraisal scale [12]. A sample item was “the current economic and employment situation would hinder any achievements I might have”. The Cronbach’s alpha for this measure in our study was 0.89.

*Threat Appraisal*. For threat appraisal, we adapted a three-item scale developed by Feldman et al. (2014) [50], which was also used in the study of Tuckey et al. (2015) [15]. The items were “I feel that the current economic and employment situation would be a negative experience for me”, “I feel that the current economic and employment situation would result in negative outcomes”, and “I feel that the current economic and employment situation would have a negative impact on me”. Evidently, these items accurately reflect the definition of threat appraisal as the perception of potential loss and harm, as delineated in the transactional model of stress [1]. However, as Petriglieri (2011) has pointed out [51], the most profound threat posed by a stressor is the harm to the value, meaning, or enactment of a person’s self-identity. Therefore, to capture the self-threat component, we adapted three items from Deichmann and Baer’s (2023) identity threat scale [52]. These items were “the current economic and employment situation makes me doubt (1) my ability to cope; (2) whether I still have a chance to succeed; and (3) whether I have irreplaceable value in myself”. Taken together, threat appraisal was measured with six items. The Cronbach’s alpha was 0.91, indicating high internal consistency for this measure.

### 4.2. Results

To validate the factor structure of the CHTA framework, we constructed a three-factor model and tested it using CFA with maximum likelihood estimation in Mplus 8.3. The three-factor model demonstrated a satisfactory fit with the data ($\chi_{\left(74\right)}^{2}$ = 127.74, *p* < 0.01, CFI = 0.96, TLI = 0.95, RMSEA = 0.068, 90% CI = [0.048, 0.088], SRMR = 0.050).

**Figure 1:**
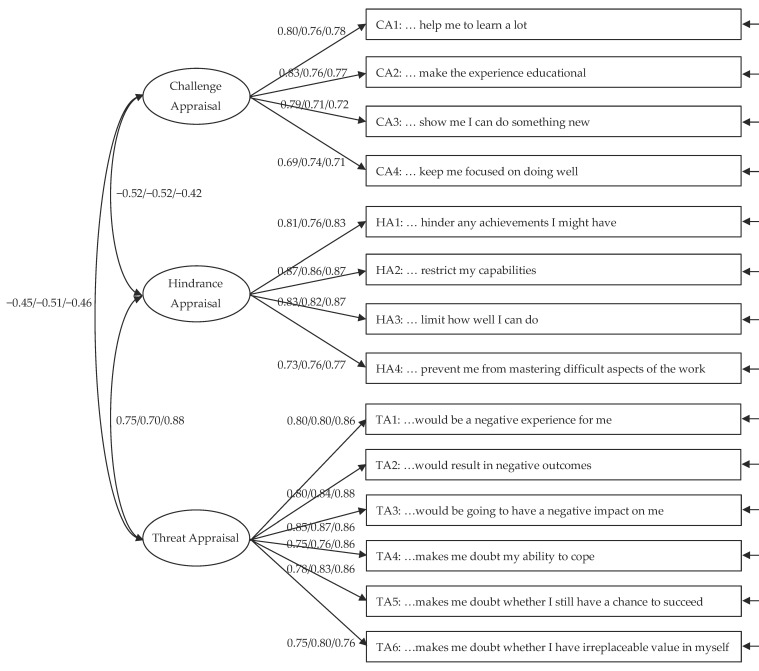
Results of confirmatory factor analyses (CFAs) for the three-factor models of the challenge–hindrance–threat appraisal (CHTA) framework across Studies 1–3. Standardized factor loadings are presented. All factor loadings are significant at the 0.001 level. The first, second, and third figures by each arrow line correspond to item factor loadings or latent correlations in Study 1, Study 2, and Study 3, respectively.

Figure 1 illustrates the factor loading of each item to its presupposed latent construct and the correlations between the three latent constructs in the three-factor model. As depicted in Figure 1, all factor loadings were statistically significant at the 0.001 level and higher than 0.50. No modification indices were identified for any of the cross-loadings, indicating that each item uniquely represented its respective latent structure. Consequently, the postulated three-factor structure was supported.

### 4.3. Study 1: Brief Discussion

In Study 1, we examined the factor structure of the CHTA using a sample of Chinese employees from various industries. The findings indicate that the three appraisal constructs in the CHTA framework are relatively independent from each other. Furthermore, the reliability of the items within the constructs was satisfactory, aligning with our expectations. Based on these results, we collected survey data to further explore other psychometric properties of the CHTA framework.

## 5. Study 2: Discriminant, Convergent, and Predictive Validity

Study 2 was conducted with three primary objectives. First, we sought to replicate the internal factor structure observed in Study 1 through CFA using a larger sample. This replication provides further evidence for the scale’s construct validity [53].

Second, we aimed to assess the discriminant and convergent validity of the CHTA through examining its relationship with the related constructs. To test its discriminant validity, we selected two personal trait constructs associated with stress appraisal: cognitive appraisal style [54] and achievement goal orientation [55]. Cognitive appraisal style, as measured using Skinner and Brewer’s (2002) cognitive appraisal scale (CAS) [54], refers to an individual’s consistent way of evaluating the personal significance of events. The threat appraisal subscale captures a tendency to focus on potential damage to self-esteem and social identity resulting from negative evaluations, while the challenge appraisal subscale emphasizes expectations of success and confidence in achieving goals. The distinction between them lies in the focus of evaluation: threat appraisals concentrate on the negative implications of stressors, whereas challenge appraisals center around positive outcomes and the ability to overcome challenges. Meanwhile, in accordance with the framework of achievement goal orientations, our study incorporated three achievement goal orientations: performance-approach goal orientation, learning goal orientation, and performance-avoidance orientation. Performance-approach goal orientations are characterized by a desire to achieve positive judgments of competence. Individuals with high performance-approach goal orientations are driven by achievement motivation, fear of failure, and high competence expectations, striving to outperform others and demonstrate excellence. On the other hand, performance-avoidance orientations revolve around the goal of avoiding negative judgments of competence. Individuals with performance-avoidance orientations are motivated by fear of failure and have low competence expectations, aiming to evade negative judgments and potential failure. Finally, learning orientations reflect an individual’s inherent interest in personal growth tasks, drawing from the trait–state anxiety relationship [56] and previous research on achievement goal orientation [57]. We hypothesized that challenge, hindrance, and threat appraisals would exhibit relationships that are related to, but separate from, these two constructs.

Third, we further explored the extent to which these three types of stress appraisals differ from each other in terms of their associations with significant workplace outcomes. Existing literature has shown that different types of stress appraisals have varying effects on employees’ work state and overall well-being [2,11,30,58]. To capture the different affective and attitudinal reactions triggered by various stress appraisals (see [15,54] for more details), we included job-related affects [59] and job insecurity [60] as the proximal outcomes.

According to the four-quadrant model of core effect presented by Warr et al., four emotions were considered: anxiety, enthusiasm, depression, and comfort [59]. These emotions correspond to different levels of emotional activation and valence. The study revealed that high activation of positive emotions (e.g., enthusiasm) was more strongly associated with positive work behaviors than low activation of positive emotions (e.g., comfort). Conversely, low activation of negative emotions (e.g., depression) was more strongly associated with negative work behaviors than high activation of negative emotions (e.g., anxiety). This insight contributes to the construction of our subsequent nomological network of CHTA. In addition, two types of job insecurity were examined: cognitive job insecurity and emotional job insecurity. Cognitive job insecurity refers to the awareness of the possibility of job loss or loss of benefits, while emotional job insecurity refers to the worries or emotional distress associated with these potential losses [60]. Understanding the differences between these two forms is crucial for gaining insight into the nuanced effects of stress appraisals on the perceived job security of employees and their subsequent attitudes and behaviors.

### 5.1. Methods

#### 5.1.1. Participants and Procedures

For Study 2, another independent sample was collected among full-time employees from various companies in China. The survey was conducted on the same online platform used in Study 1, ensuring participant anonymity, that informed consent was obtained, and that compensation was provided for participation. Due to the inclusion of additional variables, the survey required approximately 15 min to complete. To uphold data quality and counter inattentive responses, we embedded three attention check questions within the survey. Out of 667 employees invited, 468 valid responses were obtained after excluding those who did not pass the attention check. Among the valid sample (*N* = 468), 303 (64.7%) were female, ranging in age from 19 to 57 years, with a mean age of 32.97 years (*SD* = 7.18).

#### 5.1.2. Measures

*Challenge, Hindrance, and Threat Appraisal*. The same scales as in Study 1 were utilized to measure challenge appraisal, hindrance appraisal [12], and threat appraisal [15,52]. The respective Cronbach’s alpha values were 0.83, 0.87, and 0.92.

*Cognitive Appraisal Styles*. The trait style of cognitive appraisal was assessed using the CAS developed by Skinner and Brewer (2002) [54]. The *threat appraisal* subscale comprises 10 items, with an example item being “I worry what other people will think of me even when I know that it does not make any difference”. The *challenge appraisal* subscale comprises 8 items, with one sample item being “I often think about what it would be like if I do very well”. In this study, the threat subscale had a Cronbach’s alpha of 0.80, while the challenge subscale had a value of 0.94.

*Achievement Orientation*. The scale developed by Elliot and Church (1997) [55] was used to measure achievement orientation. This scale consists of 18 items that are further divided into 3 subscales. The *performance approach goal orientation* subscale comprises six items, with an example item being “It is important to me to do better than the other employees”. In this study, the Cronbach’s alpha was 0.90. The *learning goal orientation* subscale comprises six items, with one example item being “I want to learn as much as possible from this job”. The Cronbach’s alpha for this subscale was 0.82. Finally, the *performance avoidance orientation* subscale comprised six items, with one example item being “I often think to myself, what if I do badly in this job?” The Cronbach’s alpha for this subscale was 0.85.

*Job-related Affects*. We assessed four core work-related emotions (i.e., *anxiety*, *enthusiasm*, *depression*, and *comfort*) using a scale developed by Warr et al. (2014) [59]. The respective Cronbach’s alpha values for the four subscales were 0.94, 0.92, 0.92, and 0.93.

*Job Insecurity*. Huang et al. (2012) [60] originally developed a scale to measure both *cognitive job insecurity* and *affective job insecurity*. The items of cognitive job insecurity assess the levels of uncertainty and ambiguity that individuals perceive regarding their future job and career security. On the other hand, affective job insecurity measures an individual’s emotional distress or concern about losing their job or benefits. Lin and Bai (2022) recently modified this scale by including four items for each type of job insecurity, and they demonstrated that the adapted version has strong psychometric properties [61]. Therefore, we employed this adapted version to measure job insecurity; an example of a cognitive job insecurity question is “How certain are you about whether your job skills will be of value and usefulness in 6 months?”. All items were reverse-coded and rated on a scale from 1 “very uncertain” to 5 “very certain”. The internal consistency reliability of the scale was calculated to be 0.80. An example of an affective job insecurity question is “I am concerned that this company may terminate my employment at any time”. In this study, the Cronbach’s alpha value was found to be 0.94. All the masures for key variables in Study 2 are included in Appendix B.

*Control Variables*. Following the prior literature [15,37], we controlled for demographic variables, such as gender, age, education, and tenure, that may influence stress appraisals in the analyses to rule out alternative explanations.

In addition to demographic variables, we supplemented our analysis with three variables that may influence employee stress appraisal as control variables: (1) *job sector*, distinguishing between the public sector, characterized by government ownership and service orientation, and the private sector, defined by private ownership and profit motives; (2) *industry prospects*, categorized as “sunrise” for industries experiencing growth and innovation or “sunset” for those facing decline or stability with limited potential for expansion; and (3) *industry impact*, measuring the extent to which an individual’s industry is affected by the current economic and employment situation, indicating how susceptible the industry is to prevailing market conditions and employment trends.

### 5.2. Results

#### 5.2.1. Confirmative Factor Analysis

Similar to Study 1, we employed CFA to evaluate the construct validity of the challenge–hindrance–threat appraisal scale. In the hypothesized three-factor model, the three appraisals were expected to be correlated with each other but independent, and the data fit indices were deemed acceptable ($\chi_{\left(74\right)}^{2}$ = 208.51, *p* < 0.001, CFI = 0.97, TLI = 0.96, RMSEA = 0.062, 90% CI = [0.052, 0.072], SRMR = 0.031). Conversely, the two-factor and one-factor models exhibited poor fit to the data (refer to Table 2). The superiority of the three-factor model over the two-factor and one-factor models was further confirmed. Thus, the hypothesized three-factor structure was once again supported.

#### 5.2.2. Convergent and Discriminant Validity

We assessed convergent and discriminant validity using composite reliability (CR), average variance extracted (AVE), and shared variance [62,63]. According to Hair et al. (2009) [64], convergent validity is established when the CR is equal to or exceeds 0.70, and it is considered more satisfactory when it is accompanied by an AVE no less than 0.50. For discriminant validity between two factors, the AVE estimate for each factor should be greater than the square of their correlation.

We conducted CFA for an eight-factor model that included three types of stress appraisals, two trait appraisal styles, and three types of goal orientations as separate latent constructs. The eight-factor model demonstrated a good fit with the data ($\chi_{\left(1147\right)}^{2}$ = 2861.19, *p* < 0.001, CFI = 0.89, TLI = 0.88, RMSEA = 0.057, 90% CI = [0.054, 0.059], SRMR = 0.079). Based on the results of the CFA, we calculated the CR and AVE to assess the convergent validity. Table 3 shows that the CR values for each type of stress appraisal (0.83, 0.87, and 0.92) were above the threshold of 0.70, indicating adequate convergent validity. Similarly, the AVE values for each type of stress appraisal (0.55, 0.63, and 0.67) were above the threshold of 0.50, further supporting the convergent validity of the model.

For the assessment of discriminant validity, we compared the AVE of each stress appraisal with its shared variances with those of other constructs. As shown in Table 3, the AVE of each stress appraisal was greater than its shared variances with other appraisals (ranging from 0.26 to 0.49), providing further support for the three-factor model of stress appraisals. Additionally, the AVEs of hindrance and threat appraisals exceeded their shared variances with two trait appraisal styles and three types of achievement goal orientations, indicating satisfactory discriminant validity. However, the AVE of challenge appraisal was greater than its shared variance for most variables, except for trait challenge appraisal style or learning orientation.

#### 5.2.3. Predictive Validity

The key aspect of our three-factor model of stress appraisal is determining whether threat appraisal is distinct from challenge and hindrance appraisals. To explore this issue, we followed the approach suggested by Hunsley and Meyer (2003) by conducting a series of hierarchical multiple regressions [65]. This approach allowed us to assess whether threat appraisal contributes additional variance in predicting job-related affects and job insecurity beyond challenge and hindrance appraisals. In step 1, demographic variables (e.g., gender, age), trait appraisal styles, and goal orientations were included as control variables. Step 2 introduces challenge and hindrance appraisals and, in the final step, threat appraisal is added. The predictive validity of the threat appraisal was evaluated based on the changes in R-squared of the model between steps 2 and 3, where all three appraisals were included. As indicated in Table 4, the regression coefficients for threat appraisal in predicting each of the four job-related effects and the two job insecurities were found to be significant in the final step, along with the incremental R-squared gain with the addition of threat appraisal. Specifically, when predicting enthusiasm (M2, $∆R^{2}$ = 0.131, *p* < 0.01) and comfort (M4, $∆R^{2}$ = 0.159, *p* < 0.01), the regression coefficients for the other two appraisals became non-significant once threat appraisal was added. This evidence strongly suggests that threat appraisal has predictive validity beyond that of challenge and hindrance appraisals in explaining stress-related outcomes within the CHTA framework.

### 5.3. Study 2 Brief Discussion

In summary, using another sample from China, Study 2 demonstrated that the CHTA had satisfactory psychometric properties in terms of reliability, construct validity, and discriminant and convergent validity. The three appraisal constructs in the CHTA (i.e., challenge, hindrance, and threat appraisals) exhibited moderate correlations but were independent of each other. Additionally, consistent with previous research [56,57], the CHTA was clearly differentiated from two cognitive appraisal styles (i.e., trait challenge appraisal and trait threat appraisal) and three achievement goal orientations (i.e., approach, learning, and avoidance goal orientations). Moreover, when predicting stress-related outcomes (i.e., four types of work-related affects and two types of job insecurities in our study), threat appraisal had significantly greater explanatory power than challenge and hindrance appraisals, thus providing strong support for the CHTA. In other words, threat appraisal was conceptually and empirically distinct from challenge and hindrance appraisals, and the latter had a substantial incremental effect on stress-related outcomes.

However, similar to most existing research, Study 2 was based on cross-sectional data, which limited our ability to build a comprehensive and rigorous nomological network of the CHTF. Therefore, we decided to conduct a three-wave study using a new sample to further explore the relationships between the three appraisals and the attitudes and behaviors of individuals in the workplace, as well as to investigate the underlying mechanisms involved.

## 6. Study 3: Differential Effects of Cognitive Appraisals on Work Well-Being and Behaviors

The objective of Study 3 was to examine the associations between the three stress appraisals (challenge, hindrance, and threat) and various work-related proximal and distal outcomes, with a focus on uncovering their underlying mechanisms.

In the current era of swiftly evolving economic and employment landscapes, workplace stress exerts profound influences on the well-being and behavior of employees. Post-COVID-19, the normalization of remote work, the rise of gig economies, and the emergence of shared employee organizational forms have increasingly blurred traditional organizational boundaries and impaired hierarchical communications, presenting employees with a new set of challenges that are garnering the attention of researchers [66]. The advent of artificial intelligence (AI) has introduced novel challenges to job roles and required skills, with employees’ attitudes towards AI and its implications for work outcomes and psychological health becoming critical topics that require exploration [67,68,69]. Even in the absence of new technological environments, the global economic slowdown and fierce employment competition have led to excessive workloads and performance pressures, further impacting employees on a personal level [37,70]. Consequently, how individuals respond to stressors, the cognitive processes involved, and the subsequent effects on workplace-related outcomes, as well as how organizations can effectively intervene through strategic measures, have become key focal points for both organizational managers and researchers.

Since the proposal of the cognitive appraisal theory of stress, extensive research has been conducted on the processes and outcomes experienced by individuals dealing with stressors. Within the workplace context, stress appraisals have been found to significantly influence work attitudes (e.g., organizational commitment, work engagement, work passion, job satisfaction), work behaviors (e.g., creative behaviors, organizational citizenship behaviors, counterproductive behaviors, incivility), physical and psychological well-being (e.g., anxiety, depression, burnout, insecurity, psychological disengagement), and work–family relationships (e.g., work–family conflict). Different types of appraisals are often associated with different outcomes [2], and stressors appraised as hindrances or threats tend to lead to dysfunctional behaviors, while stressors appraised as challenging tend to promote functional behaviors [37]. For instance, in the study of Webster et al., no relationship was observed between challenging appraisals and psychological stress, dissatisfaction, or turnover intention; on the other hand, employees who appraised stressors as hindrances reported higher levels of dissatisfaction and turnover intention [13]. Tuckey et al. expanded on this by including threat appraisals in their study [15]. Their findings demonstrated statistically significant differences between the threat, hindrance, and challenge appraisals of employees, which were correlated with expected stressors and well-being. Specifically, threats were associated with role conflict and anxiety, hindrances with organizational constraints and fatigue, and challenges with skill demands and enthusiasm.

However, despite the progress made, there are still gaps in previous research that need to be addressed. First, there is a scarcity of studies based on the challenge–hindrance–threat appraisal framework, and those that do exist within this framework have predominantly focused on the impact of short-term emotions and attitudes, neglecting a comprehensive examination of both proximal and distal outcomes related to work. Second, as mentioned earlier, there are numerous studies directly linking specific stressors to specific cognitive appraisals, and a significant amount of research categorizes stressors into challenge and hindrance stressors. In our opinion, the validity of such an approach is open to discussion, as it represents the researcher’s cognitive evaluation of the stressor, rather than the individual’s evaluation. This divergence from the core viewpoint of the cognitive appraisal of stress theory—which emphasizes the examination of different cognitive appraisal processes in different individuals [71,72]—hampers the generalization of previous findings regarding the differential impact of various appraisals across stressful situations.

Based on the accumulating evidence for the challenge–hindrance framework and the transactional model of stress [2,11,30,58], it is anticipated that distinct cognitive appraisals of stress will manifest in varied impacts on workplace states and behaviors. Specifically, we expect that challenge appraisals could enhance work engagement and mitigate emotional exhaustion, while hindrance and threat appraisals would have detrimental effects on these outcomes. Furthermore, extensive research has indicated that work engagement is a precursor to proactive behaviors such as learning and development initiatives, whereas emotional exhaustion is a key predictor of counterproductive behaviors such as turnover intentions [73,74,75].

Therefore, in order to examine the differential effects and mediating mechanisms of the three appraisals on work-related proximal and distal outcomes, we selected representative variables associated to work attitude and work behavior; namely, work engagement, emotional exhaustion, learning behavior, and turnover intention. We propose that the three cognitive appraisals have different effects on learning behavior and turnover intention through the mediating effects of work engagement and emotional exhaustion. We anticipate that challenge appraisal will positively influence work engagement, which, in turn, will promote learning behaviors, thereby creating a virtuous cycle of personal and professional development. Conversely, hindrance and threat appraisals, through increasing emotional exhaustion, may lead to a decline in work engagement and an increase in turnover intentions, establishing a vicious cycle that could undermine individual well-being and organizational performance. Therefore, we developed two hypotheses:

**Hypothesis** **1.** *Three cognitive appraisals will have distinct effects on learning behavior, and such effects are mediated by work engagement. Specifically, challenge appraisal has a positive indirect effect on learning behaviors through work engagement (H1a), hindrance appraisal has a negative indirect effect on learning behaviors through work engagement (H1b), and threat appraisal has a negative indirect effect on learning behaviors through work engagement (H1c).*

**Hypothesis** **2.** *The three appraisals have distinct effects on turnover intention, and such effects are mediated by emotional exhaustion. Specifically, challenge appraisal has a negative indirect effect on turnover intention through emotional exhaustion (H2a), hindrance appraisal has a positive indirect effect on turnover intention through emotional exhaustion (H2b), and threat appraisal has a positive indirect effect on turnover intention through emotional exhaustion (H2c).*

In essence, our hypotheses are grounded in the premise that cognitive appraisals serve as a pivotal link between stressors and their outcomes, with work engagement and emotional exhaustion acting as critical mediators. This study aims to bridge the existing gaps in the literature by providing a more nuanced understanding of how challenge, hindrance, and threat appraisals differentially impact work-related proximal and distal outcomes through the proposed mediating mechanisms.

### 6.1. Methods

#### 6.1.1. Participants and Procedures

For Study 3, full-time employees were recruited from different companies in China to participate in a three-wave survey with a one-week lag. We utilized the Credamo platform, which, similar to the widely-used Amazon Mechanical Turk (MTurk), offers a “sample library” feature that allows researchers to import respondents from a previous survey wave into the sample library for multi-period tracking. This feature, along with the platform’s automatic matching of responses based on each user’s unique user ID across multiple waves, ensured coherent tracking of participants over time. Furthermore, we ensured participant anonymity, obtained informed consent, and committed to compensating participants for each wave of the survey. At Time 1 (T1), 499 employees responded. At Time 2 (T2), 325 of the 499 initial participants completed the second survey, with a retention rate of 65.13%. At Time 3 (T3), the final questionnaire was sent to the 325 employees who completed the second survey, and 311 employees responded, with a retention rate of 95.69%. In the final sample (*N* = 311), 220 (70.7%) participants were female, ranging in age from 20 to 60 years, with a mean age of 33.89 years (*SD* = 7.13) and a mean organizational tenure of 6.93 years (*SD* = 5.26).

#### 6.1.2. Measures

*Challenge, Hindrance, and Threat Appraisal*. The stress appraisal measures used in T1 were consistent with those employed in Studies 1 and 2. All three appraisals demonstrated high reliability, as evidenced by the following Cronbach’s alpha values: challenge appraisal (α = 0.83), hindrance appraisal (α = 0.90), and threat appraisal (α = 0.94).

*Work Engagement.* At T2, work engagement was measured using Houle et al.’s (2022) revised 9-item scale [76]. An example item was “I exert my full effort in my job”. The scale exhibited good internal consistency, with a Cronbach’s alpha of 0.88.

*Emotional Exhaustion*. At T2, we also measured emotional exhaustion using a 3-item scale developed by Watkins et al. (2015) [77], including “I feel exhausted when I think about having to face another day on the job”. In this study, the Cronbach’s alpha was 0.90.

*Learning Behavior*. At T3, we used the eight-item scale of Bezuijen et al. (2010) to measure how frequently an individual engages in various learning behaviors, which has been demonstrated to have sufficient psychometric properties in the Chinese context [61]. A sample item was “Within my task responsibilities, I actively look for methods to improve my work”. The scale demonstrated good internal consistency, with a Cronbach’s alpha of 0.81.

*Turnover Intention*. At T3, we also used the 3-item scale of Schaubroeck, Lam, and Xie to measure turnover intention [78]. A sample item was “I often think about quitting my job”. In this study, the Cronbach’s alpha was 0.72. All the measures for key variables in Study 3 are included in Appendix B.

*Control Variables*. Consistent with Study 2, we measured the individual’s gender, age, education, tenure, job sector, industry prospects, and industry impact.

### 6.2. Results

#### 6.2.1. Descriptive Statistics and Confirmative Factor Analysis

Means, *SD*s, α coefficients, and bivariate correlations for the Study 3 variables are presented in Table 5.

CFA was conducted to assess the construct validity of the challenge–hindrance–threat appraisal scale following the approach employed in Studies 1 and 2. The three-factor model achieved a good fit to the data ($\chi_{\left(74\right)}^{2}$ = 127.91, *p* < 0.001, CFI = 0.98, TLI = 0.98, RMSEA = 0.048, 90% CI = [0.034, 0.062], SRMR = 0.032), with no suggested modifications. Figure 1 illustrates factor loadings ranging from 0.60 to 0.81, all of which are significant at the 0.001 level, indicating strong construct validity.

#### 6.2.2. Differential Effects of Challenge, Hindrance, and Threat Appraisals

**Figure 2:**
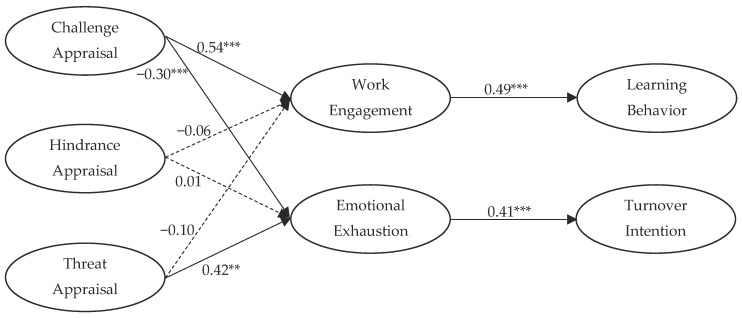
Structural equation modeling (SEM) results for appraisals, work engagement, emotional exhaustion, learning behavior, and turnover intention (Study 3); ** *p* < 0.01; *** *p* < 0.001. Circles indicate that all four variables were constructed as latent variables in the SEM. For clarity, the indicators of each latent variable are intentionally omitted from the figure. The solid lines in the figure represent significant paths, while the dotted lines indicate non-significant paths.

Structural equation modeling (SEM) was employed to examine the influences of challenge, hindrance, and threat appraisals on learning behavior and turnover intention, mediated by work engagement and emotional exhaustion. The mediating model demonstrated an acceptable fit to the data ($\chi_{\left(474\right)}^{2}$ = 1063.55, *p* < 0.001, CFI = 0.90, TLI = 0.89, RMSEA = 0.063, 90% CI = [0.058, 0.068], SRMR = 0.052). Specifically, challenge appraisal exhibited a positive association with work engagement (γ = 0.54, *t* = 4.09, *p* < 0.01) and a negative association with emotional exhaustion (γ = −0.30, *t* = −2.72, *p* < 0.01). In contrast, hindrance appraisal demonstrated no significant relationship with either work engagement (γ = −0.06, *t* = −0.13, *p* = 0.90) or emotional exhaustion (γ = 0.01, *t* = 0.01, *p* = 0.98). Threat appraisal was not significantly associated with work engagement (γ = −0.05, *t* = −0.10, *p* = 0.91) but was significantly positively related to emotional exhaustion (γ = 0.42, *t* = 3.94, *p* < 0.01). Furthermore, work engagement exhibited a positive association with learning behavior (γ = 0.49, *t* = 2.96, *p* < 0.01), while emotional exhaustion displayed a positive association with turnover intention (γ = 0.41, *t* = 2.84, *p* < 0.01). Based on the path coefficients, we further examined the indirect effects of 5000 replicates using the bootstrap method (Hayes, 2013). The bootstrap results revealed significant indirect effects of challenging appraisals on learning behaviors through work engagement (*b* = 0.199, *SE* = 0.113, 95%CI = [0.055, 0.439]) and on turnover intention through emotional exhaustion (*b* = −0.129, *SE* = 0.067, 95%CI = [−0.290, −0.034]). However, the indirect effects of hindrance appraisal on both learning behavior (*b* = −0.014, *SE* = 0.248, 95%CI = [−0.134, 0.096]) and turnover intention (*b* = −0.002, *SE* = 0.131, 95%CI = [−0.149, 0.137]) were not significant. Threat appraisal had a non-significant indirect effect on learning behavior through work engagement (*b* = −0.010, *SE* = 0.241, 95%CI = [−0.144, 0.084]) but a significant indirect effect on turnover intention through emotional exhaustion (*b* = 0.112, *SE* = 0.132, 95%CI = [0.004, 0.319]). The SEM results are depicted in Figure 2.

#### 6.2.3. Supplement Analysis

To further explore the complexities of the relationships between stress appraisals, work engagement, emotional exhaustion, learning behaviors, and turnover intention, we conducted additional analyses to examine the cross-pathway effects within the framework.

We added cross-paths to the SEM in order to investigate the effects of stress appraisals on turnover intention through work engagement and learning behavior through emotional exhaustion. This was considered because individuals who are engaged in their work are less likely to consider leaving their current job [79,80,81,82], while employees who experience emotional exhaustion may also have reduced learning at work [83,84,85].

The fit indices for the mediator model that included cross-paths remained satisfactory ($\chi_{\left(472\right)}^{2}$ = 1057.324, *p* < 0.001, CFI = 0.90, TLI = 0.89, RMSEA = 0.063, 90% CI = [0.058, 0.068], SRMR = 0.098). As a complement to Study 3, we found that, in the two newly added cross-paths, work engagement significantly influenced turnover intention (γ = −0.22, t = −2.30, *p* < 0.05), while emotional exhaustion did not significantly predict learning behavior (γ = −0.04, t = −0.32, *p* = 0.75). The coefficients and significance for all paths are reported in Appendix C.

The results of the supplementary analyses demonstrated that the relationships between the three stress appraisals and work engagement, emotional exhaustion, learning behavior, and turnover intention remained consistent with the inclusion of cross-paths. In other words, the three appraisals still had differential effects on work attitudes and behaviors.

### 6.3. Study 3 Brief Discussion

The findings of Study 3 present compelling evidence for differentiating threat appraisals from challenge and hindrance appraisals. Through a three-wave sample of full-time employees, we validated the three-factor structure of the CHTA based on the CFA results. This demonstrates the consistent application of the CHTA factor structure across various samples. Thus, the results of Study 3 support our assertion that the integrated three-factor framework of stress appraisal (challenge, hindrance, and threat) surpasses the individual challenge–hindrance or challenge–threat frameworks.

Furthermore, Study 3 revealed distinct predictions associated with each appraisal in terms of employee attitudes and outcomes. Challenge appraisals positively influence employee engagement in learning behaviors and decrease turnover intentions through fostering work engagement. Conversely, hindrance appraisals have no impact on work attitudes or outcomes. Finally, threat appraisals increase employee turnover intentions through emotional exhaustion. It is crucial to emphasize the distinction between threat appraisal and the other two appraisals as well as the prominence of threat appraisal in predicting employee attitudes and outcomes in the workplace. This understanding is vital for effective stress management in the current economic and employment climate.

## 7. Discussion

Through integrating both the transactional theory of stress [1] and the challenge–hindrance framework [2,10,11], and building upon the work of Tuckey et al. [15], we developed an integrated challenge–hindrance–threat framework for stress appraisal. This framework captures an individual’s evaluation of a stressor in terms of the risk of personal loss and self-harm (threat) as well as the facilitation (challenge) or hindrance of personal benefit (hindrance). Using three independent samples, we developed a three-factor model for the CHTA framework (Study 1), cross-validated its factor structure, tested the psychometric properties of the measures for three cognitive appraisals (Study 2), and revealed the differential effects of three cognitive appraisals on proximal and distal outcomes (Study 3). As a result, our study answered LePine’s (2022) call that “it may be crucial to examine challenge, hindrance, and threat demands and appraisals in order to fully understand the nature of these stressors and their effect on performance, growth, and well-being” [16] (p. 236).

### 7.1. Implications for the Challenge–Hindrance–Threat Appraisal Framework

The cognitive appraisal theory of stress posits that the effects of work stressors on employees and organizations vary based on the types of stressors and the appraisals that individuals make of them. Although threats have received insufficient attention in the context of challenge–hindrance stressor research [15], their significance in the present challenging economic and employment landscape cannot be overstated. Through integrating the transactional theory of stress [1] and building upon the work of Tuckey et al. (2015) [15], we developed an integrated challenge–hindrance–threat framework for stress appraisal. This framework captures an individual’s evaluation of a stressor in terms of the risk of personal loss and self-harm (threat) as well as facilitation (challenge) or hindrance of personal benefit (hindrance).

The integrated framework presented in the current research was supported by the results of three independent and progressive studies. Using a multi-sample strategy, we measured all three appraisals in terms of reliability (internal consistency) and validity (construct validity, convergent validity, and discriminant validity), cross-validating the psychometric properties of the challenge–hindrance–threat appraisal framework. Specifically, in all three studies, the three-factor model was found to have a convincing factor structure, and the measurements of each appraisal also had good reliability. Furthermore, in Study 2, the convergent validity, discriminant validity, and predictive validity were tested. The results indicated that (1) the CHTA framework is distinct from both cognitive appraisal styles (challenge and threat) and achievement goal orientations (approach, learning, and avoidance), and (2) threat appraisal has greater explanatory power than challenge and hindrance appraisals in predicting stress-related outcomes, specifically job impact and job insecurity. That is, when examining the relevant outcome variables, the three appraisals are independent and clearly distinguishable from each other. Finally, Study 3 further provided convincing evidence for distinguishing threat appraisal from challenge and hindrance appraisals using longitudinal data. Our findings regarding the impact of the challenge appraisal framework on employee attitudes and outcomes indicate that the three appraisals have distinct effects: particularly hindrance and threat appraisals. Threat appraisals are associated with negative attitudes and outcomes in the workplace, while hindrance appraisals showed no correlation. Therefore, the results of the three studies support our view that the comprehensive three-factor framework for stress appraisal (challenge, hindrance, and threat) transcends the individual challenge–hindrance or challenge–threat frameworks, echoing recent scholarly attention to threat appraisal and calls for an integrated framework [14,15,16].

Additionally, this article addresses recent calls for an examination of the diverse cognitive appraisal processes triggered by the same stressors [19,20], rather than categorizing stressors [86,87] or anchoring specific stressors to specific types of appraisal [71], as in traditional approaches to challenges, hindrances, or even threats [15,16]. Through adopting a measurement strategy similar to that of Webster et al. (2011), we highlight the importance of diverse appraisals of the same stressors by individuals, aligning with research on cognitive appraisal of stress and transactional theory and the core perspective of stress [71,72]. Our research provides empirical evidence for individuals differently appraising the same stressor. Individuals may appraise current forms of economic employment as a challenge, hindrance, and threat, which can be independent of each other or may exist simultaneously. This is consistent with previous findings on goal difficulty [45], performance pressure [37,88], and other work-related stressors [18,36], indicating that our approach and results will not be questioned in relation to other stressors.

In summary, the results from our three studies consistently demonstrate that individuals, when faced with the same stressor—namely, the current economic and employment situation—may simultaneously generate a variety of cognitive appraisals, including challenge, hindrance, and threat. These appraisals are not only distinct from one another but also exhibit clear independence, particularly highlighting the differentiation between hindrance and threat appraisals, which have often been conflated in the past. This study encourages future researchers to further investigate the meaning, antecedents, and consequences of stress appraisals within specific stressful situations, utilizing the challenge–hindrance–threat framework as a foundation for their investigations.

### 7.2. Differential Effects of Three Cognitive Appraisals on Work-Related Outcomes

Based on the accumulating evidence of the challenge–hindrance framework and the transactional model of stress [2,11,30,58], it is anticipated that the distinct cognitive appraisals of stress will manifest in varied impacts on workplace states and behaviors. Recent research on challenge, hindrance, and threat appraisals have also shown that challenge appraisals are associated with activated positive emotions, hindrance appraisals are associated with burnout, and threat appraisals are associated with anxiety and anger [15]. According to the four-quadrant model of core affects presented by Warr et al. (2014), Study 2 focused on four types of affect: high-activation positive affect (enthusiasm), low-activation positive affect (comfort), high-activation negative affect (anxiety), and low-activation negative affect (depression). In addition, we also considered two types of job insecurity: emotional job insecurity and cognitive job insecurity. Our findings indicated that threat appraisal has a higher and more unique explanatory power than challenge and hindrance appraisals when predicting proximal outcomes that include the four types of affect and two types of job insecurity. This provides preliminary evidence for examination of the differential effects and mediating mechanisms of the three appraisals on work-related proximal and distal outcomes.

More importantly, in Study 3, we further selected representative variables of proximal and distal outcomes to comprehensively examine the differential effects of the three appraisals under the same stressor and examined their mediating mechanisms, which have not been addressed in previous studies. Specifically, we expect that challenge appraisal could result in favorable outcomes that enhance work engagement and mitigate emotional exhaustion, while hindrance and threat appraisals would be expected to have dysfunctional relationships with these outcomes. The results of Study 3 indicate that the three different appraisals—challenge, hindrance, and threat—have varying effects on work-related attitudes and outcomes when faced with the same stressor. Specifically, challenge appraisals enhance the learning behaviors of employees and decrease their willingness to leave through promoting work engagement. In contrast, hindrance appraisals do not exert any significant influence on attitudes or outcomes, while threat appraisals heighten the willingness to leave through emotional exhaustion.

Through introducing representative variables from work-related proximal and distal outcomes into the challenge–hindrance–threat appraisal framework, we provide a comprehensive perspective to explain the differential effects of these three cognitive appraisals of the same stressor on the individual outcomes of employees in the workplace and their mediating mechanisms. In doing so, our research extends existing literature in three important areas. First, regarding the literature focused on the challenge–threat and challenge–hindrance frameworks, we emphasize the important and unique role of threat appraisal in predicting workplace outcomes and individual psychological well-being related to stress, as threats have not been given much attention and were often mixed up with hindrances in the past [15,16]. Second, within the challenge–hindrance–threat framework, we chose to examine three appraisals of individuals under the same stressor, rather than categorizing stressors [86,87] or anchoring specific stressors to specific types of appraisal [71]—which is a questionable traditional approach—in response to recent calls [19,20]. Finally, in response to the call of Lepine et al. (2022), we comprehensively examined the differential effects and mediating mechanisms of three appraisals on the proximal and distal work-related outcomes. The positive influence of challenge appraisal on work engagement and learning behaviors suggests that, when employees perceive stressors as opportunities for growth, it can lead to a virtuous cycle of development. This cycle fosters a proactive approach to work, enhances skill acquisition, and contributes to the overall positive climate within the organization. In contrast, the non-significant impact of hindrance appraisals on work attitudes and outcomes indicates that, while these stressors may be recognized, they do not necessarily dictate the employee’s behavioral or attitudinal response. This could be attributed to individual differences in coping strategies or organizational support systems that mitigate the adverse effects of hindrance stressors. As for the negative influence of threat appraisals, their role in increasing emotional exhaustion and turnover intentions is a particularly critical finding. This implies that perceiving stressors as threats can initiate a vicious cycle that erodes employee well-being and commitment. The heightened sense of vulnerability and potential loss associated with threat appraisals can undermine an employee’s resilience and job satisfaction, making it imperative for organizations to address such perceptions proactively. These findings provide new insights and evidence for expanding stress research.

### 7.3. Practical Implications

Considering the current economic climate and its impact on employment, our study has significant practical implications. In the modern dynamic economic landscape, the role of threat appraisals in the workplace has become increasingly critical [89,90]. Our findings carry significant implications for managerial practice, human resource strategies, and employees.

From a managerial standpoint, our research highlights the importance of understanding that employees may perceive the same stressor differently. The implications for management are clear: fostering an environment that encourages employees to view stressors as challenges rather than threats is crucial. Managers should focus on providing the support and resources necessary to help employees appraise stress positively, thereby enhancing work engagement and reducing emotional exhaustion. Therefore, organizations must proactively address threat appraisals within their workforce to mitigate these negative effects. This necessitates developing an awareness of these perceptions and implementing strategies to address them. Moreover, employees require support in navigating the complexities of the modern workplace. Equipping them with the ability to reframe stressors as opportunities rather than threats is essential. This can be accomplished through fostering a growth mindset, where challenges are seen as opportunities to learn and develop new skills, thereby promoting personal and professional growth. Additionally, employees should possess effective stress management tools, such as mindfulness techniques, time management strategies, and the capacity to seek social support from colleagues.

Additionally, from an HR perspective, this study underscores the need for proactive human resources strategies that address the cognitive appraisal of stress. HR should develop programs aimed at enhancing employees’ resilience and stress management capabilities. In doing so, HR can play a pivotal role in cultivating a workforce that is better equipped to navigate the complexities of the modern workplace, thus reducing the prevalence of threat appraisals and their negative outcomes. Training managers to recognize signs of threat appraisals among their team members enables them to provide appropriate support and resources. This includes offering personalized feedback, career development opportunities, and creating a safe space for employees to express their concerns. Furthermore, human resource managers should actively cultivate a supportive work culture that encourages open communication, making employees feel heard and valued. In this way, human resource managers can empower employees to reframe their perceptions of stressors, transforming potential threats into catalysts for positive change and innovation.

Finally, from the employee’s perspective, it is essential for individuals to take ownership of their stress response. Employees should actively engage in the resources and training provided by their organizations to better manage their work-related stress. Through adopting strategies that promote a positive outlook on stressors and leveraging available tools for stress reduction, employees can improve their work engagement and mitigate the risk of emotional exhaustion and turnover intentions.

In conclusion, the current study stresses the collective responsibility of managers, HR, and employees in shaping the cognitive appraisal of stressors. A collaborative approach is key to transforming the workplace into an environment that supports well-being, engagement, and productivity. Through viewing the current economic climate and employment situation as an opportunity for growth and providing the necessary support, organizations can enhance their employees’ psychological well-being and job performance, ultimately fostering a more productive and harmonious workplace.

### 7.4. Limitations and Future Directions

Although we provided consistent evidence for the psychometric properties of the CHTF in three separate studies using diverse samples, the current study has several limitations. The first limitation pertains to the use of self-reported data. Given our focus on the cognitive appraisal process of stress—which is largely subjective in nature—this study relied on self-reported data from employed individuals in all three studies. This approach aligns with previous research on cognitive appraisal theories of stress. Additionally, in Study 3, a three-stage longitudinal study was conducted to mitigate potential common method bias. However, despite these efforts, the causal relationships between the three stress appraisals and stress-related attitudes and outcomes have not been conclusively validated. Therefore, future studies should build upon our findings through the employment of more rigorous research designs, such as experimental intervention studies, in order to determine the causal effects of stress appraisals on stress-related attitudes and outcomes.

Another limitation arises from our limited exploration of other organizations and work contexts. First, our study focused specifically on the stressor of a challenging economic development and employment situation. Although the challenging economic development and employment situation is a critical stressor in the current global climate, it represents only one of many stressors that employees may encounter. The emergence of artificial intelligence (AI) and its impact on job roles and required skills is a notable example [67,68]. Second, but more insightfully, our study’s focus on the differential effects of cognitive appraisals did not allow us to fully explore the boundary conditions of these effects. The transactional model of stress suggests that cognitive appraisal processes are influenced by both individual and environmental factors [1]. On the one hand, individual traits can significantly affect the cognitive appraisal process [91]; for example, resilience reflects an individual’s capacity to adapt to stressful environments and can protect against the negative impacts of stressors [92]. When performance pressure serves as a stressor, resilient employees are not only effective at filtering out stressor-related information but also appraise it positively, making them more likely to make challenge appraisals and less likely to make hindrance appraisals [37]. Similarly, individuals with a proactive personality tend to evaluate stressors more positively [93]. On the other hand, situational factors also play a crucial role in the cognitive appraisal process. For example, a supportive organizational culture may encourage employees to view changes as challenges to be embraced, while a more hierarchical and less supportive culture might lead to increased hindrance and threat appraisals. Leadership behaviors can also set the tone for how stressors are appraised; leaders who model resilience and optimism can inspire employees to adopt a similar outlook. Recent studies have found that organizational support [94] and a favorable participative climate [95] can weaken employees’ negative cognitive appraisals of stressors. Through examining both individual and situational factors, future studies can delineate the boundary conditions that influence the appraisal process and identify the circumstances in which different appraisals are most salient. This expanded perspective will contribute to a deeper understanding of the complex interplay between stressors, appraisals, and outcomes, ultimately informing more effective stress management and intervention strategies in organizations.

Finally, our study is limited by the narrow spectrum of outcomes related to stress that we were able to examine. The primary objective of this study was to assess the need to distinguish between the three types of stress appraisals and, particularly, the distinctiveness of threat appraisals compared to the other two. Consequently, this study was restricted to a specific set of outcome variables. It is recommended that future research investigate the differential effects of these appraisals across a broader range, including behavioral aspects and tangible job outcomes. Recent empirical evidence on stressors has suggested that, in a rapidly changing economic and employment landscape, certain specific stressors may elicit unique behavioral responses from employees. For example, the shift to remote work and the increase in workload have become more common, and these stressors can lead to a variety of outcomes, such as reduced safety behavior or even increased counterproductive work behaviors [66,70]. Understanding the relationship between these specific stressors and employee behavior is crucial for developing targeted interventions and managing workplace stress more effectively. Through broadening the scope of outcome variables to include these and other relevant behaviors, future research can provide a more nuanced understanding of how different stress appraisals influence a wide array of employee responses. This expanded perspective will contribute to a more comprehensive view of the long-term implications of stress appraisals on behavior and performance within organizational settings.

## 8. Conclusions

Overall, this research provided compelling psychometric evidence to support the challenge–hindrance–threat appraisal (CHTA) framework, introduced to examine workplace stress. This integrated work is not only useful for academic researchers but also has practical value for practitioners. In addition, our study elucidated the differential effects and mediating mechanisms of three types of stress cognitive appraisals (challenge, hindrance, and threat) on work-related proximal and distal outcomes, emphasizing the importance of distinguishing the three appraisals—particularly threat appraisals—in stress management to improve employee well-being and organizational efficiency.

## Figures and Tables

**Table 1:** Overview of Studies 1, 2, and 3.

Studies	Objectives	Samples	Analytical Strategies
Study 1	(1) To generate measurement items for CHTA (2) To examine the factor structure of CHTA	Cross-sectional survey (*N* = 224)	Confirmatory factor analysis (CFA)
Study 2	(1) To recheck the factor structure of CHTA (2) To examine the discriminant, convergent, and predictive validity of CHTA	Cross-sectional survey (*N* = 468)	Composite reliability (CR), average variance extracted (AVE), and hierarchical multiple regressions
Study 3	(1) To examine the differential effects of the three appraisals on work-related outcomes (2) To investigate the underlying mechanisms	Three-wave survey (*N* = 311)	Structural equation modeling (SEM)

Note: CHTA = the challenge–hindrance–threat appraisal framework.

**Table 2:** Results of model fit indicators for the three-factor, two-factor, and one-factor models of the challenge–hindrance–threat appraisal scale (Study 2).

Model	$\chi^{2}$	df	RMSEA	CFI	SRMR
Three-factor model	208.51	74	0.97	0.96	0.03
Two-factor model (combining CA and HA)	639.39	76	0.86	0.83	0.08
Two-factor model (combining CA and TA)	687.82	76	0.85	0.82	0.09
Two-factor model (combining HA and TA)	643.72	76	0.86	0.83	0.07
One-factor model (combining CA, HA, and TA)	1084.60	77	0.75	0.70	0.10

Note: CA = challenge Challenge appraisalAppraisal; HA = hindrance Hindrance appraisalAppraisal; TA = threat Threat appraisalAppraisal; RMSEA = Root Mean Square Error of Approximation; CFI = Comparative Fit Index; SRMR= Standardized Root Mean Square Residual.

**Table 3:** Descriptive statistics, psychometric properties, and latent correlations among variables (Study 2).

	Mean	*SD*	α	CR	AVE	1	2	3	4	5	6	7	8
(1) Challenge appraisal	4.01	0.82	0.83	0.83	0.55	/	0.27	0.26	0.72	0.30	0.16	0.60	0.23
(2) Hindrance appraisal	2.86	1.06	0.87	0.87	0.63	−0.52 ***	/	0.49	0.18	0.30	0.00	0.10	0.28
(3) Threat appraisal	3.21	1.07	0.92	0.92	0.67	−0.51 ***	0.70 ***	/	0.18	0.40	0.00	0.10	0.38
(4) Trait challenge appraisal style	4.03	0.59	0.80	0.79	0.34	0.85 ***	−0.43 ***	−0.42 ***	/	0.33	0.31	0.79	0.24
(5) Trait threat appraisal style	2.71	1.00	0.94	0.94	0.63	−0.55 ***	0.55 ***	0.63 ***	0.58 ***	/	0.00	0.19	0.82
(6) Performance-approach orientation	3.97	0.80	0.90	0.80	0.59	0.40 ***	−0.06	−0.06	0.56 ***	−0.04	/	0.34	0.00
(7) Learning orientation	4.17	0.60	0.82	0.82	0.44	0.78 ***	−0.31 ***	−0.31 ***	0.89 ***	−0.44 ***	0.58 ***	/	0.14
(8) Performance-avoidance orientation	2.99	0.94	0.85	0.85	0.49	−0.48 ***	0.53 ***	0.62 ***	−0.49 ***	0.90 ***	0.05	−0.38 ***	/

*** *p* < 0.001. α = Cronbach’s α coefficient; CR = composite reliability; AVE = average variance extracted. Latent correlations are included below the diagonal, and shared variances are presented above the diagonal.

**Table 4:** Hierarchical regression results for mindsets of creativity and intelligence (Study 2).

	DV: Job-Related Affect												DV: Job Insecurity					
	Anxiety			Enthusiasm			Depression			Comfort			Cognitive Job Insecurity			Affective Job Insecurity		
	M1a	M1b	M1c	M2a	M2b	M2c	M3a	M3b	M3c	M4a	M4b	M4c	M5a	M5b	M5c	M6a	M6b	M6c
Gender	−0.08	−0.06	−0.05	0.11	0.07	0.06	−0.01	0.03	0.04	0.07	0.05	0.04	−0.17 *	−0.14	−0.13	−0.02	0.01	0.01
Age	−0.01	−0.01	−0.01	0.01	0.01	0.00	−0.01	−0.01	−0.01	0.01	0.01	0.01	−0.01	−0.00	−0.00	−0.01	−0.01	−0.01
Education	−0.09	−0.03	0.01	0.18 **	0.13 *	0.10	−0.16 **	−0.10	−0.08	0.05	0.01	−0.03	−0.08	−0.04	−0.02	−0.18 **	−0.13 *	−0.10
Tenure	0.00	0.00	0.00	−0.00	−0.01	−0.01	0.00	0.01	0.01	−0.00	−0.00	−0.01	−0.01	−0.00	−0.00	−0.00	0.00	0.00
Job sector	−0.04	0.00	0.06	−0.04	−0.06	−0.11	0.06	0.09	0.13	−0.02	−0.05	−0.10	0.05	0.06	0.09	−0.02	0.01	0.05
Industry prospects	−0.61 **	−0.38 **	−0.17 *	0.73 **	0.48 **	0.30 **	−0.64 **	−0.37 **	−0.18 *	0.62 **	0.45 **	0.26 **	−0.49 **	−0.29 **	−0.19 **	−0.31 **	−0.08	0.06
Industry impact	0.47 **	0.27 **	0.13 **	−0.38 **	−0.20 **	−0.08	0.48 **	0.24 **	0.15 **	−0.33 **	−0.18 **	−0.07	0.43 **	0.28 **	0.22 **	0.64 **	0.46 **	0.37 **
Challenge appraisal		−0.09	0.02		0.31 **	0.22 **		−0.31 **	−0.24 **		0.12 *	0.04		−0.25 **	−0.21 **		−0.18 **	−0.12 *
Hindrance appraisal		0.47 **	0.18 **		−0.29 **	−0.05		0.43 **	0.23 **		−0.30 **	−0.06		0.23 **	0.10*		0.36 **	0.17 **
Threat appraisal			0.63 **			−0.53 **			0.43 **			−0.54 **			0.28 **			0.43 **
*R* ^2^	0.278 **	0.450 **	0.630 **	0.272 **	0.407 **	0.538 **	0.314 **	0.539 **	0.628 **	0.229 **	0.331 **	0.490 **	0.307 **	0.428 **	0.481 **	0.312 **	0.434 **	0.513 **
∆ *R* ^2^		0.171 **	0.181 **		0.135 **	0.131 **		0.226 **	0.089 **		0.102 **	0.159 **		0.121 **	0.053 **		0.122 **	0.078 **
*F*	25.349 ***	41.561 ***	77.950 ***	24.591 ***	34.931 ***	53.189 ***	30.012 ***	59.559 ***	77.147 ***	19.489 ***	25.185 ***	43.995 ***	29.100 ***	38.009 ***	42.275 ***	29.806 ***	39.082 ***	48.059 ***
∆F		71.219 ***	223.625 ***		52.025 ***	129.389 ***		112.192 ***	109.018 ***		35.029 ***	143.007 ***		48.262 ***	46.610 ***		49.534 ***	73.317 ***

* *p* < 0.05; ** *p* < 0.01; *** *p* < 0.001.

**Table 5:** Descriptive statistics, psychometric properties, and latent correlations among variables (Study 3).

	Mean	*SD*	α	1	2	3	4	5	6	7
(1) Challenge appraisal (T1)	4.10	0.80	0.83	/						
(2) Hindrance appraisal (T1)	2.82	1.16	0.90	−0.36 ***	/					
(3) Threat appraisal (T1)	3.00	1.16	0.94	−0.40 ***	0.81 ***	/				
(4) Work engagement (T2)	4.19	0.59	0.88	0.51 ***	−0.32 ***	−0.35 ***	/			
(5) Emotion exhaustion (T2)	2.20	1.04	0.94	−0.39 ***	0.45 ***	0.52 ***	0.61 ***	/		
(6) Learning behavior (T3)	4.31	0.48	0.81	0.45 ***	−0.14 *	−0.22 ***	0.58 ***	−0.36 ***	/	
(7) Turnover intention (T3)	1.68	0.68	0.72	−0.34 ***	0.24 ***	0.29 ***	−0.43 ***	0.46 ***	−0.52 ***	/

* *p* < 0.05; *** *p* < 0.001. α = Cronbach’s α coefficient.

**Figure A1:**
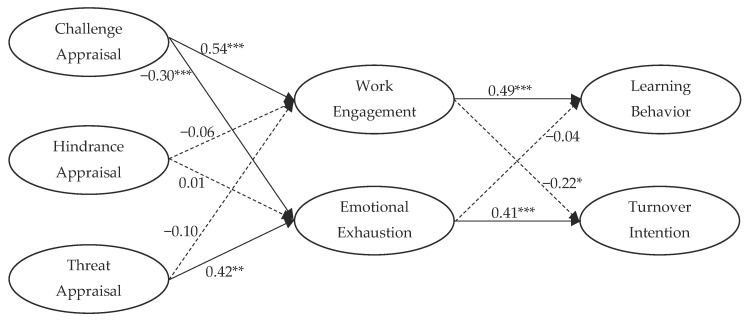
Structural equation modeling (SEM) results for appraisals, work engagement, emotional exhaustion, learning behavior, and turnover intention (supplement analysis). * *p* < 0.05; ** *p* < 0.01; *** *p* < 0.001. Circles indicate that all four variables were constructed as latent variables in the SEM. For clarity, the indicators of each latent variable are intentionally omitted from the figure. The solid lines in the figure represent significant paths; the dotted lines indicate non-significant paths.

## Data Availability

The data that support the findings of this study are available in [Science Data Bank] at https://doi.org/10.57760/sciencedb.10237.

## Appendix A. Measures for Challenge, Hindrance, and Threat Appraisals

**Table A1:** Scales for challenge, hindrance, and threat appraisals used in Studies 1, 2, and 3.

**Challenge Appraisal** (1) The current economic and employment situation would help me to learn a lot. (2) The current economic and employment situation would make the experience educational. (3) The current economic and employment situation would show me I can do something new. (4) The current economic and employment situation would keep me focused on doing well. **Hindrance Appraisal** (5) The current economic and employment situation would hinder any achievements I might have. (6) The current economic and employment situation would restrict my capabilities. (7) The current economic and employment situation would limit how well I can do my job. (8) The current economic and employment situation would prevent me from mastering difficult aspects of the work. **Threat Appraisal** (9) I feel that the current economic and employment situation would be a negative experience for me. (10) I feel that the current economic and employment situation would result in negative outcomes. (11) I feel that the current economic and employment situation would have a negative impact on me. (12) The current economic and employment situation makes me doubt my ability to cope. (13) The current economic and employment situation makes me doubt whether I still have a chance to succeed. (14) The current economic and employment situation makes me doubt whether I have irreplaceable value in myself.

Note. A five-point Likert-type scale, 1 = strongly disagree, 5 = strongly agree.

## Appendix B. Measures for Key Variables in Studies 2 and 3

**Table A2:** Cognitive appraisal styles scale (Study 2, Skinner & Brewer, 2002 [54]).

**T** **hreat** **Appraisal Style** (1) I worry that I will say or do the wrong things. (2) I worry about the kind of impression I make. (3) I am concerned that others will find fault with me. (4) Sometimes I think that I am too concerned with what other people think of me. (5) I feel that difficulties are piling up so that I cannot overcome them. (6) I lack self-confidence. (7) I worry what other people will think of me even when I know that it doesn’t make any difference. (8) I am concerned that others will not approve of me. (9) I worry about what other people may be thinking about me. (10) I feel like a failure. **Challenge Appraisal Style** (11) I tend to focus on the positive aspects of any situation. (12) I often think about what it would be like if I do very well. (13) I believe that most stressful situations contain the potential for positive benefits. (14) Overall, I expect that I will achieve success rather than experience failure. (15) In general, I look forward to the rewards and benefits of success. (16) A challenging situation motivates me to increase my efforts. (17) In general, I anticipate being successful at my chosen pursuits, rather than expecting to fail. (18) I look forward to opportunities to fully test the limits of my skills and abilities.

Note. A five-point Likert-type scale, 1 = strongly disagree, 5 = strongly agree.

**Table A3:** Achievement orientation scale (Study 2, Elliot & Church, 1997 [55]).

**Performance Approach Goal Orientation** (1) It is important to me to do better than the other students. (2) My goal in this class is to get a better grade than most of the students. (3) I am striving to demonstrate my ability relative to others in this class. (4) I am motivated by the thought of outperforming my peers in this class. (5) It is important to me to do well compared to others in this class. (6) I want to do well in this class to show my ability to my family, friends, advisors, or others. **Learning Goal Orientation** (7) I want to learn as much as possible from this class. (8) It is important for me to understand the content of this course as thoroughly as possible. (9) I hope to have gained a broader and deeper knowledge of psychology when I am done with this class. (10) I desire to completely master the material presented in this class. (11) In a class like this, I prefer course material that arouses my curiosity, even if it is difficult to learn. (12) In a class like this, I prefer course material that really challenges me so I can learn new things. **Performance Avoidance Goal Orientation** (13) I often think to myself, “What if I do badly in this class?” (14) I worry about the possibility of getting a bad grade in this class. (15) My fear of performing poorly in this class is often what motivates me. (16) I just want to avoid doing poorly in this class. (17) I’m afraid that if I ask my TA or instructor a “dumb” question, they might not think I’m very smart. (18) I wish this class was not graded.

Note. A five-point Likert-type scale, 1 = strongly disagree, 5 = strongly agree.

**Table A4:** Job-related affects scale (Study 2, Warr et al., 2014 [59]).

**Anxiety (high-activation unpleasant affect)** (1) The current situation makes me feel anxious. (2) The current situation makes me feel nervous. (3) The current situation makes me feel tense. (4) The current situation makes me feel worried. **Enthusiasm (high-activation pleasant affect)** (5) The current situation makes me feel enthusiastic. (6) The current situation makes me feel excited. (7) The current situation makes me feel inspired. (8) The current situation makes me feel joyful. **Depression (low-activation unpleasant affect)** (9) The current situation makes me feel dejected. (10) The current situation makes me feel depressed. (11) The current situation makes me feel despondent. (12) The current situation makes me feel hopeless. **Comfort (low-activation pleasant affect)** (13) The current situation makes me feel at ease. (14) The current situation makes me feel calm. (15) The current situation makes me feel laid back. (16) The current situation makes me feel relaxed.

Note. A five-point Likert-type scale (1 = strongly disagree, 5 = strongly agree).

**Table A5:** Job insecurity scale (Study 2, Huang et al., 2012 [60]).

**Cognitive Job Insecurity** (1) How certain are you about the prospects for your future career development and self-improvement? (Reversed) (2) How certain are you that you will have opportunities for promotion or self-development in the next few years? (Reversed) (3) How certain are you that your knowledge and skills will continue to be relevant over the next five years? (Reversed) (4) How certain are you about whether your job skills will be of value and usefulness in 6 months? (Reversed) 1 = very uncertain, 5 = very certain.
**Affective Job Insecurity** (5) I am tense about maintaining my current job employment status. (6) I feel uneasy about my chances for remaining with this company. (7) I am scared by the thought of losing my job. (8) I am worried that this company will fire me any time.

Note. A five-point Likert-type scale, 1 = strongly disagree, 5 = strongly agree.

**Table A6:** Work engagement scale (Study 3, Houle et al., 2022 [76]).

**Physical Engagement** (1) I give my all in my job. (2) I put maximum effort into doing my job well. (3) I devote a lot of energy to my job. **Emotional Engagement** (4) I am enthusiastic about my work. (5) I am interested in my work. (6) I feel excited about my work. **Cognitive Engagement** (7) When working, I am fully absorbed. (8) When working, I am very focused. (9) When working, I concentrate deeply.

Note. A five-point Likert-type scale, 1 = strongly disagree, 5 = strongly agree.

**Table A7:** Emotional exhaustion scale (Study 3, Watkins et al., 2015 [77]).

(1) I feel emotionally drained from my work. (2) I feel burned out from my work. (3) I feel exhausted when I think about having to face another day on the job.

Note. A five-point Likert-type scale, 1 = strongly disagree, 5 = strongly agree.

**Table A8:** Learning behavior scale (Study 3, Bezuijen et al., 2010 [61]).

(1) I spend time following a course or educational program. (2) I am working to extend my knowledge and skills. (3) I perform learning tasks that are not part of my job. (4) I spend time planning and realizing my career. (5) I go to my supervisor to discuss how I can make progress. (6) Within my task responsibilities, I actively look for methods to improve my work. (7) Within my job, I look for activities from which I can learn. (8) I continually learn new skills for my job.

Note. A five-point Likert-type scale, 1 = strongly disagree, 5 = strongly agree.

**Table A9:** Turnover intention scale (Study 3, Schaubroeck et al., 2000 [78]).

(1) I often think about quitting my job. (2) There is a strong possibility that I will resign from my position in the next few months. (3) Considering all factors, I really want to leave my current organization.

Note. A five-point Likert-type scale, 1 = strongly disagree, 5 = strongly agree.
